# Characteristics of Online Health Care Services From China’s Largest Online Medical Platform: Cross-sectional Survey Study

**DOI:** 10.2196/25817

**Published:** 2021-04-15

**Authors:** Xuehan Jiang, Hong Xie, Rui Tang, Yanmei Du, Tao Li, Jinsheng Gao, Xiuping Xu, Siqi Jiang, Tingting Zhao, Wei Zhao, Xingzhi Sun, Gang Hu, Dejun Wu, Guotong Xie

**Affiliations:** 1 Ping An Healthcare Technology Beijing China; 2 Ping An Healthcare and Technology Company Limited Shanghai China

**Keywords:** eHealth, internet hospital, China, online health care services, mHealth, COVID-19, digital health, app, online consultation, user experience

## Abstract

**Background:**

Internet hospitals in China are in great demand due to limited and unevenly distributed health care resources, lack of family doctors, increased burdens of chronic diseases, and rapid growth of the aged population. The COVID-19 epidemic catalyzed the expansion of online health care services. In recent years, internet hospitals have been rapidly developed. Ping An Good Doctor is the largest, national online medical entry point in China and is a widely used platform providing online health care services.

**Objective:**

This study aims to give a comprehensive description of the characteristics of the online consultations and inquisitions in Ping An Good Doctor. The analyses tried to answer the following questions: (1) What are the characteristics of the consultations in Ping An Good Doctor in terms of department and disease profiles? (2) Who uses the online health services most frequently? and (3) How is the user experience of the online consultations of Ping An Good Doctor?

**Methods:**

A total of 35.3 million consultations and inquisitions over the course of 1 year were analyzed with respect to the distributions of departments and diseases, user profiles, and consulting behaviors.

**Results:**

The geographical distribution of the usage of Ping An Good Doctor showed that Shandong (18.4%), Yunnan (15.6%), Shaanxi (7.2%), and Guangdong (5.5%) were the provinces that used it the most; they accounted for 46.6% of the total consultations and inquisitions. In terms of department distribution, we found that gynecology and obstetrics (19.2%), dermatology (17.0%), and pediatrics (14.4%) were the top three departments in Ping An Good Doctor. The disease distribution analysis showed that, except for nondisease-specific consultations, acute upper respiratory infection (AURI) (4.1%), pregnancy (2.8%), and dermatitis (2.4%) were the most frequently consulted diseases. In terms of user profiles, females (60.4%) from 19 to 35 years of age were most likely to seek consultations online, in general. The user behavior analyses showed that the peak times of day for online consultations occurred at 10 AM, 3 PM, and 9 PM. Regarding user experience, 93.0% of users gave full marks following their consultations. For some disease-related health problems, such as AURI, dermatitis, and eczema, the feedback scores were above average.

**Conclusions:**

The prevalence of internet hospitals, such as Ping An Good Doctor, illustrated the great demand for online health care services that can go beyond geographical limitations. Our analyses showed that nondisease-specific issues and moderate health problems were much more frequently consulted about than severe clinical conditions. This indicated that internet hospitals played the role of the family doctor, which helped to relieve the stress placed on offline hospitals and facilitated people’s lives. In addition, good user experiences, especially regarding disease-related inquisitions, suggested that online health services can help solve health problems. With support from the government and acceptance by the public, online health care services could develop at a fast pace and greatly benefit people’s daily lives.

## Introduction

### Background

Internet hospital is an innovative type of hospital where professional physicians provide health care services on the internet. The online health care services at these hospitals include, but not limited to, health-related consultations, disease diagnoses, medication prescription, and chronic disease management [1].

In this study, internet hospitals generally refer to all applications that provide online health services for disease consultation and treatment through information technology. Services by internet hospitals include a combination of offline medical treatment and online follow-up consultation, telemedicine, online medical consultation, and online health management. Internet hospitals are sometimes referred to worldwide as telehealth [2]. In the United States, each state’s laws, regulations, and Medicaid program policies for telehealth differ significantly. However, the common aspect they share is that most states and Washington, DC, provide reimbursement via Medicaid for some form of live video as a fee-for-service [3]. In the European Union, most countries have no formal definition of telemedicine services [4]. There is an online consultation system in South West England that allows adult patients to contact their general practitioner, but evidence indicates that the use of e-consultations is very low [5]. Compared with developed countries, telehealth could be more meaningful in developing countries because of limited medical resources and poor health care services [5,6].

Internet hospitals have been rapidly established in China in recent years [7-9]. As of May 2019, there were 158 internet hospitals in China [10], and as of October 28, 2020, there were about 900 of them operating in China [11]. The expansion of internet hospitals is due to the following five reasons.

First, health care resources are limited and unevenly distributed by geography in China [12]. By 2019, there were only 2.77 licensed physicians per 1000 people [13]; for city residents, there were 4.10 licensed physicians per 1000 people, while that number was 1.96 for rural residents [13]. There are 3-tier health care systems in China [14,15]. Primary health care providers are usually community based and are expected to play the role of general practitioner and to perform health care management. Secondary and tertiary health care providers include more specialists and focus on more complicated clinical problems. However, tertiary health care providers are mainly located in the eastern cities of China where the economies are more developed. The top 100 hospitals in China have mostly been located in big cities, such as Beijing and Shanghai, and provincial capitals [16]. As a result, residents in rural areas or western cities have had limited access to high-quality health care services [16].

Second, family doctor systems in China have been underdeveloped [17,18]. As the family doctor plays a gatekeeping role in developed countries, it is convenient for one to seek health care services for mild problems. Although the Chinese government launched a series of policies and regulations to accelerate family doctors’ contracting services during the last decades, family doctor systems are still at an early stage in China [17]. The number of general practitioners was not sufficient due to the large population, wide geographic area, and uneven distribution of health care resources. As a consequence, the effect of family doctor systems was compromised in practice [19]. With low public awareness of diseases, patients with moderate symptoms also visited the tertiary hospitals, putting more stress on health care resources. According to the China Health Statistics Yearbook 2020, tertiary hospitals covered 53.5% of patient visits in China, while primary hospitals only accounted for 6.0% [13]. Internet hospitals are easy to access and serve as a supplement to family doctors. Therefore, internet hospitals meet the population’s need for convenient access to professional medical help in China.

Third, clinical data sharing has been hindered by unconnected hospital systems. Such information islands result in patients having to endure repeated examinations when changing to a new hospital. However, with internet hospitals, patients are able to upload their existing examination results, which avoids wasting clinical resources.

Fourth, with the increasing burden of medical insurance, the Chinese government proposed the *Healthy China 2030* plan [20] and put more focus on health-driven management instead of traditional disease-driven treatment. For the increasing aged population, health care providers should intervene at the onset of chronic diseases and take advantage of artificial intelligence technology to manage the disease at the same time.

Last but not least, after the outbreak of COVID-19, offline treatment channels were blocked and the Chinese government had adopted a series of administrative measures to encourage the development of internet hospitals [21]. Unnecessary face-to-face contact was avoided with internet hospitals, providing safer and more convenient health care services than in-person, offline hospitals. As suggested by recent work, internet hospitals helped control the COVID-19 epidemic [22,23] and made access to health care services more convenient [24].

In terms of the types of initiators, internet hospitals in China can be divided into government-led, hospital-led, and enterprise-led services [8]. In 2012, the Guangdong Second Provincial General Hospital built the first internet hospital in China, which belonged to the first type of internet hospital (ie, government-led) [14]. Compared with government-led and hospital-led internet hospitals, enterprise-led internet hospitals have the advantages of stronger capabilities in market exploration and faster product iterations. Enterprises are more open to advanced technology and pay more attention to improving the efficiency of consultation.

Ping An Good Doctor has been one of the leading companies of enterprise-led internet hospitals [21]. It provides health care services via a mobile app of the same name, *Ping An Good Doctor*. Ping An Good Doctor aimed to create a one-stop, whole-process, online-to-offline service platform and integrated online health service platform with offline health services, such as private clinics, pharmacies, health checkup and test centers, and so on [25]. It was rapidly developed, as there was an urgent need for internet hospitals in China. The use of Ping An Good Doctor was nationwide. There were 346 million registered users, more than 1800 staff members working for in-house medical teams, and about 10,000 external experts by the middle of 2020. There were more than 820 million online consultations and inquisitions in total from 2014 until now, which covered a wide range of departments and diseases. The number of cumulative visits during the COVID-19 epidemic—from the period of January 20 to February 10, 2020—reached 1.11 billion [26]. Besides Ping An Good Doctor, Ali and JD are also major players in internet health care in China [27]. However, Ping An Good Doctor has the largest average daily consultation volume [28-30].

Any user with a cell phone can use the health care services provided by the Ping An Good Doctor app anytime and anywhere. Users can ask any health-related questions or seek health care services whether or not they have a specific health problem. In general, a dialogue in the absence of health problems is referred to as a consultation, while seeking clinical help for specific health problems is called an inquisition. In the latter scenario, users are expected to submit their chief complaint and other clinical materials about their health status; the physician would then make the diagnosis and provide corresponding health care services, including prescriptions if needed. Medications, if needed, would be delivered if the users chose to buy them online. All consultations and inquisitions are documented for further quality examination. At the end of the process, users are also asked to score the consultation, where 1 stands for the least satisfaction and 5 for the most satisfaction.

### Study Objectives

Despite the rapid development of internet hospitals in China, there exist limited studies examining the characteristics of the departments and diseases of internet hospitals. To fill this gap, we carried out analyses regarding the nationally utilized Ping An Good Doctor platform, aiming to provide a comprehensive description of online medical care in China. Our primary focus was on answering the following three questions: (1) What are the characteristics of the consultations and inquisitions in Ping An Good Doctor in terms of department and disease profiles? (2) Who most often use the platform’s online health services? and (3) How do users experience the online consultations of Ping An Good Doctor?

## Methods

### Data Collection

To eliminate the influence of seasonal patterns, we analyzed the online consultations and inquisitions of Ping An Good Doctor from the past year (ie, from August 2019 to August 2020). Here, consultation referred to the asking of health-related questions, while inquisition meant being treated online for health problems. For all consultations and inquisitions, four types of information were extracted: (1) demographic information about the clients, (2) health care–related variables, (3) details about the consultation process, and (4) consultation evaluations collected after the consultation finished. A detailed description of all variables involved in this study is listed in Table 1.

**Table 1:** Variables considered in this study.

Type of information and variables			Description of variables		Responses (N=113,805,518^a^), n (%)
**Demographic information**					
	Gender	Gender of the user		111,844,894 (98.3)	
	Age	Age of the user		110,502,135 (97.1)	
	Province	Province (ie, location) of the user		103,786,537 (91.2)	
**Health care–related variables**					
	Diagnosis	Diagnosis as given by the physician		81,574,890 (71.7)	
	Department	Subentity of the online hospital, such as dermatology		113,805,518 (100)	
**Details about consultation process**					
	Dialogue rounds	Number of rounds of dialogue between the user and the physician		113,805,518 (100)	
	Consultation time	Time of day when the user described their chief complaints		113,805,518 (100)	
**Consultation evaluation**					
	Satisfaction score	Scores given by the user after the consultation and/or inquisition		6,879,529 (6.0)	

^a^This value represents all consultations and inquisitions combined.

### Data Selection

**Figure 1:**
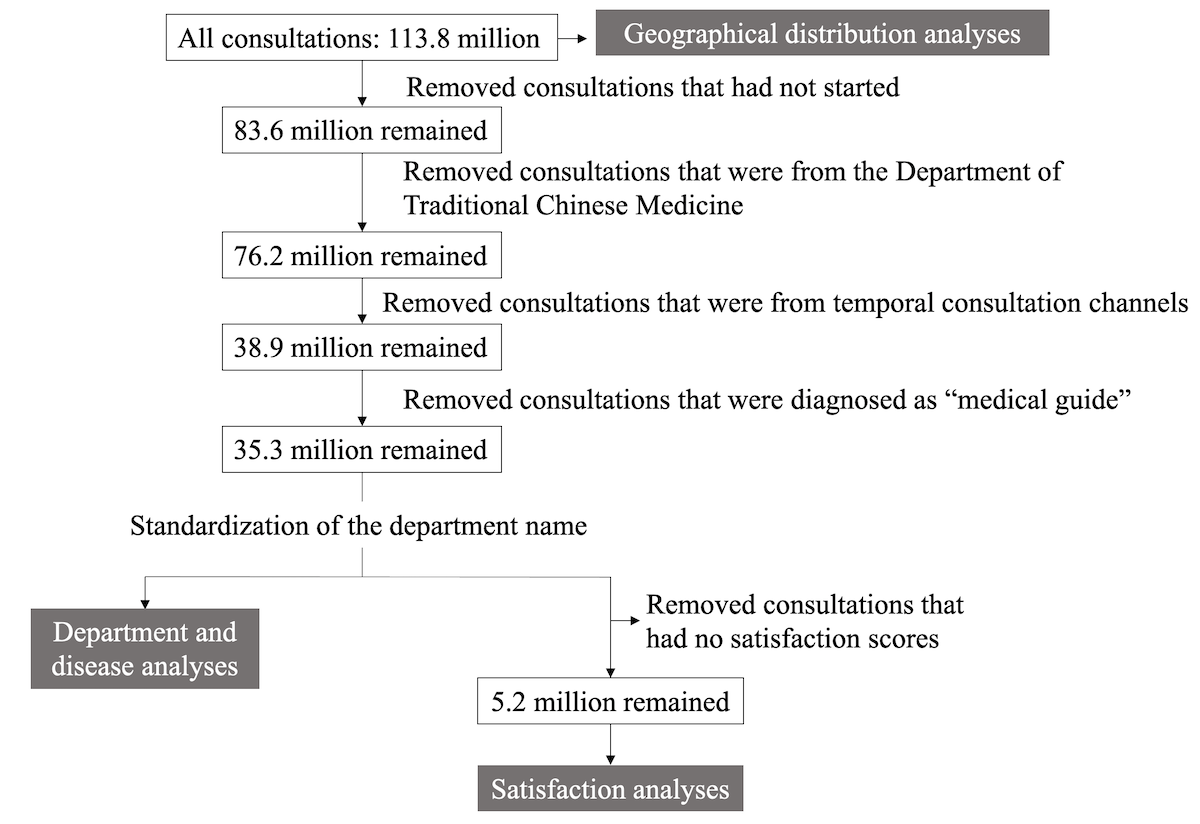
The process of data selection and the data sets that were used for different analyses.

The number of consultations and inquisitions accumulated between August 20, 2019, and August 22, 2020, was around 113.8 million. The detailed process of data selection is illustrated in Figure 1. Four types of consultations and inquisitions were excluded before the analyses of departments and diseases. First, invalid consultations, where the consultations and inquisitions had not started, were removed. Second, consultations from the Traditional Chinese Medicine (TCM) department were excluded since the diagnosis system of TCM is distinct from that of the International Classification of Diseases, Tenth Revision (ICD-10), and is poorly standardized. Third, consultations and inquisitions from some temporal consultation channels, such as the special consulting channel during COVID-19, that would bias the distributions of departments and diseases were also omitted. Lastly, we removed consultations that were labeled as “medical guide,” which was an automatic diagnosis label assigned under certain circumstances, such as the unexpected end of a consultation due to the extended nonresponse of the user. After the four data-filtering steps, there were 35.3 million consultations and inquisitions remaining. After standardization of the departments, those data were used for department- and disease-related analyses. To further analyze user satisfaction of the online consultations, data that had no satisfaction scores were filtered out. 

### Data Preprocessing

The diagnoses from the inquisitions were mainly based on the ICD-10 coding system [31], while consultations were assigned self-defined diagnosis labels, such as *general consultations*, *specific consultations*, and *lifestyle-related questions*. Here, the term *general consultations* was a vague definition referring to asking questions about any nonspecific aspect of health problems, such as how one should take exercise if one has had a stroke. *Specific consultations* referred to asking questions about a specific disease, such as diabetes and cardiovascular diseases. *Lifestyle-related questions* referred to asking questions about how to stay healthy without mentioning specific health problems, such as what tea to drink to protect eyesight.

Generally, the diagnosis labels for inquisitions and consultations were standardized as one-by-one mapping to ICD-10 or self-defined codes.

It should be noted that for 20.2% (7,140,917/35,296,169) of the consultations and inquisitions, there existed multiple diagnoses. In this case, the diagnosis distribution was calculated by splitting the multiple diagnoses of one sample into multiple samples, each sample with a distinct diagnosis. All analyses in terms of diagnoses were based on this splitting approach of diagnosis statistics, such as user experience for each diagnosis.

### Statistical Analysis

Data were extracted from the database using Structured Query Language and were preprocessed by the *pandas* library in Python 3.6 (Python Software Foundation).

## Results

### Overview of the Online Consultations and Inquisitions

The overall statistics of our data set are shown in Table 2. There were 35,296,169 valid consultations and inquisitions in total. During the study period, 12,446,838 users participated in consultations via Ping An Good Doctor. A total of 40.3% of the consultations and inquisitions were made by male users, and the mean age of the users was 27.3 years (SD 17.2). There were, on average, 7.3 (SD 5.3) rounds of conversation between users and physicians. After standardization, there were 76 departments and 1419 diseases covered in the cross section that we studied.

**Figure 2:**
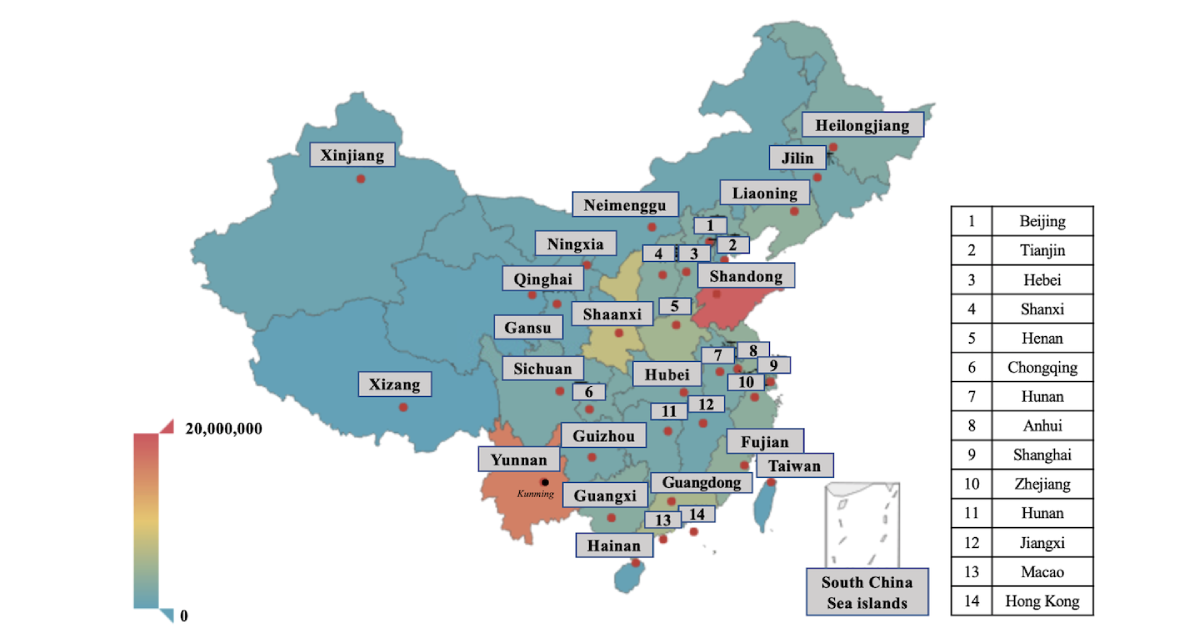
The number of consultations and inquisitions from all over China out of the original 113.8 million data points. Red dots indicate the capital city of each province and the black dot indicates the city of Kunming. Kunming had the largest volume of consultations and inquisitions in Ping An Good Doctor at the city level.

As shown in Figure 2, which was drawn using the *pyecharts* library in Python 3.6, the consultations and inquisitions came from all the provinces, municipalities, and autonomous regions of China. Shandong (19,067,141/113,805,518, 18.4%), Yunnan (16,192,209/113,805,518, 15.6%), Shaanxi (7,439,540/113,805,518, 7.2%), and Guangdong (5,672,425/113,805,518, 5.5%) were the four leading provinces and accounted for 46.6% (48,371,315/113,805,518 ) of the total consultations and inquisitions in our data set. A city-level rank was also calculated. Table 3 lists the top 10 cities in yearly visits to Ping An Good Doctor. It was interesting to note that big cities such as Beijing and Shanghai only ranked 9^th^ and 5^th^, respectively, accounting for 34.4% (1,699,251/4,932,918) and 54.7% (2,700,223/4,932,918) of the total visits, relative to the top city, Kunming.

Longitudinally, the COVID-19 epidemic activated the daily visits to Ping An Good Doctor. As shown in Table 4 [32,33], the average daily visits increased by 23.2% during the outbreak of COVID-19 in China. It was encouraging that the average daily visits increased more even after the outbreak of COVID-19 in China.

**Table 2:** Characteristics of the current data set.

Characteristic	Value
Total consultations and inquisitions, N	35,296,169
Consultations and inquisitions by male users, n (%)	14,063,178/34,904,918 (40.3)
Users of the platform, n	12,446,858
Age of users who submitted consultations and inquisitions (years), mean (SD)	27.3 (17.2)
Rounds of dialogue between physicians and users, mean (SD)	7.3 (5.3)
Departments represented in the data set, n	76
Diseases represented in the data set, n	1419

**Table 3:** Top 10 cities by visits to Ping An Good Doctor from August 2019 to August 2020.

City	Province	Relative yearly visit amount^a^
Kunming	Yunnan	1
Xi’an	Shaanxi	0.915
Yantai	Shandong	0.835
Qingdao	Shandong	0.559
Shanghai	Shanghai	0.547
Chongqing	Chongqing	0.424
Linyi	Shandong	0.410
Honghe Hani and Yi Autonomous Prefecture	Yunnan	0.385
Beijing	Beijing	0.344
Zhengzhou	Henan	0.305

^a^Values are based on the original 113.8 million data points; relative yearly visits of each city are calculated by setting the yearly visit amount by Kunming as a reference.

**Table 4:** Average daily visits to Ping An Good Doctor before, during, and after the outbreak of COVID-19 in China.

Phase	Time range	Daily visits, mean (SD)
Before the COVID-19 outbreak	August 20, 2019, to January 22, 2020	74,893.92 (5843.34)
During the COVID-19 outbreak	January 23, 2020^a^, to February 29, 2020^b^	92,246.74 (20,411.83)
After the COVID-19 outbreak	March 1, 2020, to August 22, 2020	114,898.50 (11,708.20)

^a^The city of Wuhan closed on January 23, 2020, indicating the start of the outbreak of COVID-19 in China [32].

^b^As of February 29, 2020, the number of newly diagnosed COVID-19 cases per day in China was less than 1000 [33].

### Characteristics of Departments and Diseases in Online Consultations

#### Distribution of Departments

**Figure 3:**
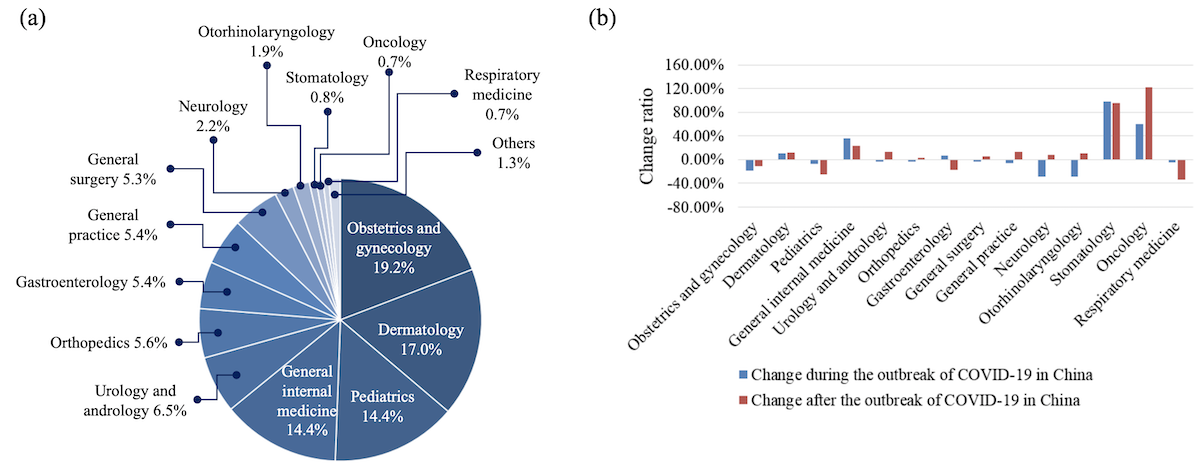
Departments represented in the consultations and inquisitions: (a) distribution of the departments; (b) change ratios of the distribution of the top 14 departments during and after the outbreak of COVID-19 in China.

Although there were 76 departments represented in our data set, about 14 (18%) departments accounted for more than 99% of the consultations and inquisitions. As shown in Figure 3 (a), the most popular department was gynecology and obstetrics, which was represented in 19.2% (5,868,172/30,579,297) of the consultations, followed by 17.0% (5,204,805/30,579,297) for the dermatology department and 14.4% (4,394,634/30,579,297) for pediatrics. We further analyzed the change ratios of distribution for the top 14 departments during and after the COVID-19 outbreak to investigate the effect of the COVID-19 epidemic on the distribution of departments. The result is shown in Figure 3 (b); for most of the departments, the change ratios were within ±10% during and after the COVID-19 outbreak. It should be noted that the fraction of consultations represented by dermatology increased by more than 10%, both during (from 15. 9% to 17.5%) and after (from 15.9% to 17.7%) the COVID-19 outbreak. In addition, the fraction of consultations represented by general internal medicine increased by 36.5% (from 11.7% to 16.0%) and 22.7% (from 11.7% to 14.4%) during and after the COVID-19 outbreak, respectively. Although the fractions of consultations represented by stomatology and oncology both had a sharp increase, their absolute fractions increased by 0.47% and 0.51% after the COVID-19 outbreak, respectively.

#### Distribution of Diseases

**Figure 4:**
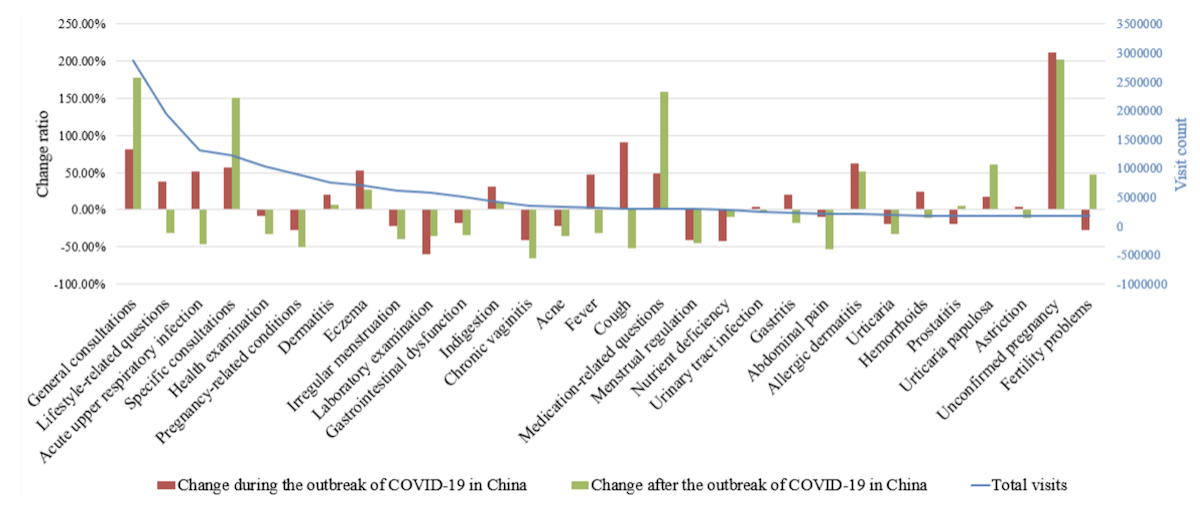
Distribution of the top 30 diseases represented in the yearly visits to Ping An Good Doctor; change ratios of the distribution of diseases during and after the outbreak of COVID-19 in China are also shown.

There were 1419 diseases represented in our data set, and the top 30 diseases accounted for 53.6% (17,911,125/33,409,879) of the total consultations. As illustrated by the blue line in Figure 4, consultations were the key components in terms of yearly visits to Ping An Good Doctor; these included *general consultations*, *lifestyle-related questions*, *specific consultations*, *health examinations*, and *pregnancy-related conditions*. The change ratios of disease distribution during and after the COVID-19 outbreak were also analyzed. As shown by the red bars in Figure 4, diseases labeled as *unconfirmed pregnancy*, *cough*, *consultations*, and *specific consultations* increased the most during the COVID-19 outbreak. In addition, *eczema* and *allergic dermatitis* also had a significant increase. To summarize, diseases that increased in popularity during the COVID-19 outbreak, as represented in consultations and inquisitions, included those with respiratory or dermatology symptoms. After the COVID-19 outbreak, the inquisitions that included respiratory symptoms decreased, while the inquisitions that included characteristics of other diseases and consultations remained.

#### Disease Profiles for the Most Popular Departments

**Figure 5:**
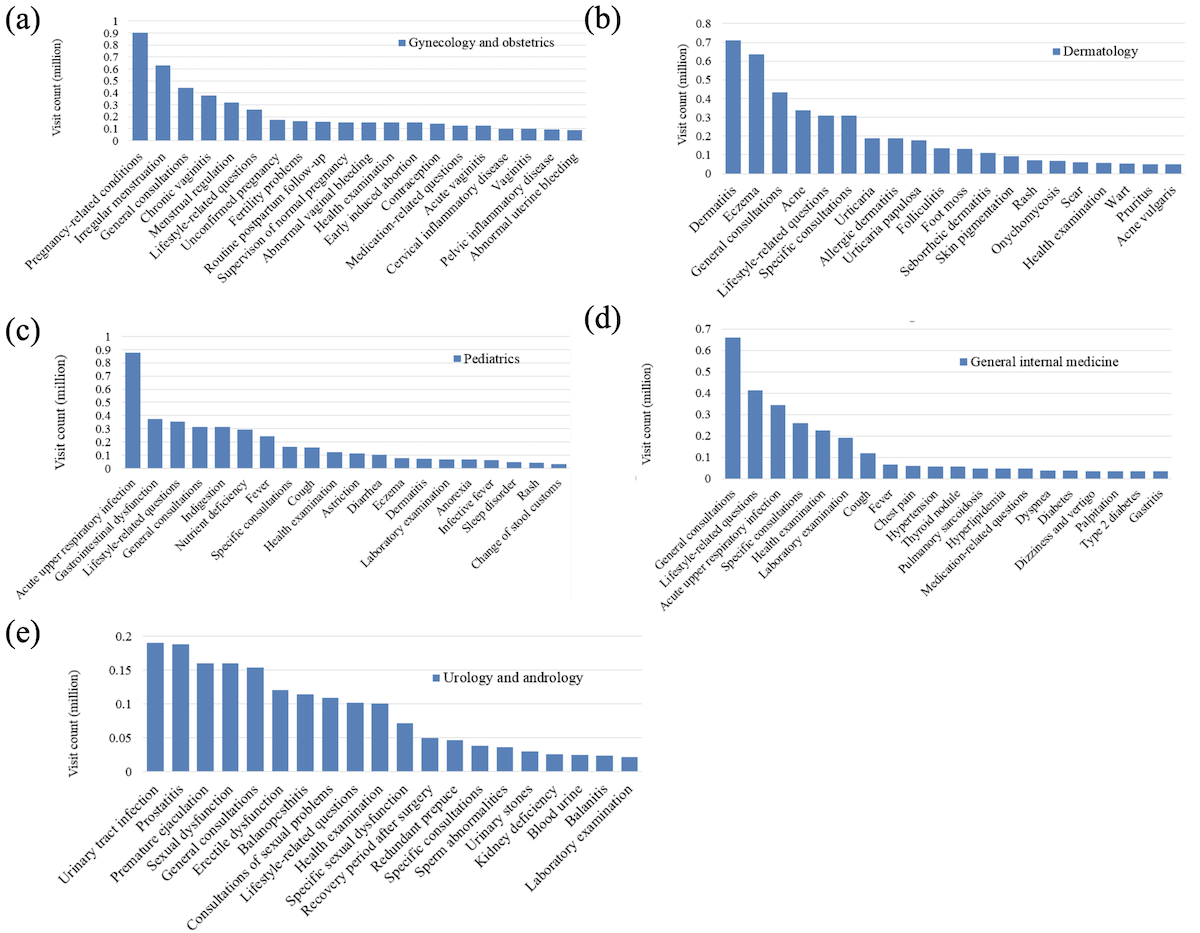
Disease profiles of the top five departments: (a) gynecology and obstetrics, (b) dermatology, (c) pediatrics, (d) general internal medicine, and (e) urology and andrology.

We further analyzed the disease profiles for the five most popular departments to see if major diseases existed in each department. The results are illustrated in Figure 5; for each department, the top 20 diseases are shown.

From Figure 5, pediatrics had the most obvious head effect regarding disease profiles, while acute upper respiratory infection (AURI) accounted for 16.5% (876,786/5,074,566) of all the consultations. In addition, urology and andrology had the least head effect regarding disease profiles, suggesting a relatively high degree of disease diversity.

### User Profiles

#### Demographic Characteristics

**Figure 6:**
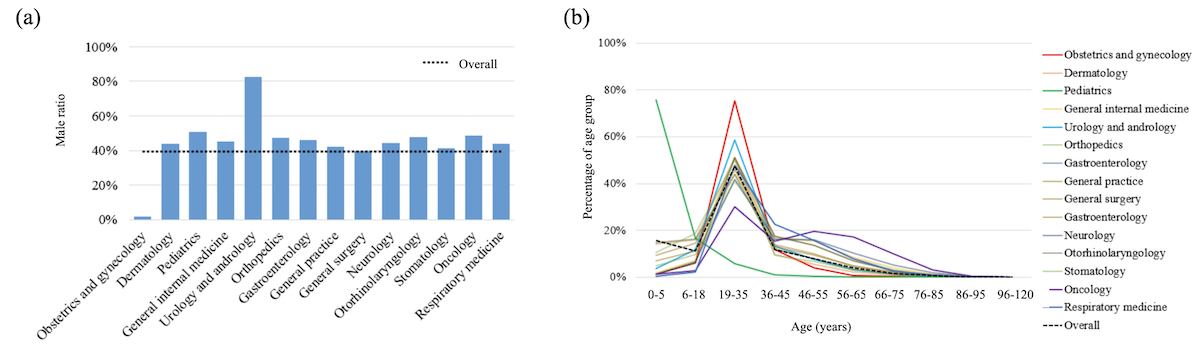
User profiles for the top 14 departments: (a) percentiles of male users by department; (b) age distribution.

To better understand the users of Ping An Good Doctor, we analyzed the user profiles for the top 14 departments. Male users accounted for 39.6% (12,098,998/30,579,308) of the consultations; this proportion varied little except for the reproductive-related departments, as shown in Figure 6 (a). For most of the departments, users aged 19 to 35 years constituted the largest proportion of users as compared with other age groups, as listed in Figure 6 (b). For pediatrics, there were many more consultations and inquisitions for children under 6 years of age than for children older than 6 years of age. For the respiratory medicine department , there was a relatively higher proportion of users aged 36 to 45 years as compared with the overall age groups. For oncology, the proportion of users in the senior age ranges increased.

#### User Behaviors

The user profiles for the top diseases had concordant observations with those for the department analyses. We further investigated user consultation behaviors with respect to different diseases; namely, the times of day when users began to seek consultations and the rounds of dialogue between users and physicians.

**Figure 7:**
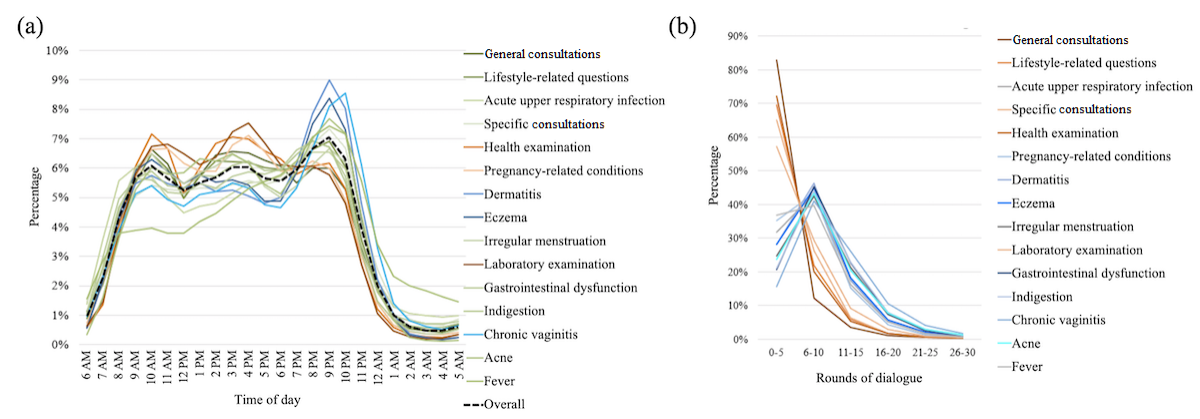
Characteristics of users' behaviors regarding the top 15 diseases: (a) distribution of times of day of consultations; (b) distribution of rounds of dialogue between users and physicians.

As shown in Figure 7 (a), there were three peaks regarding times of day for consultations; these were 10 AM, 3 PM, and 9 PM. The diseases could be roughly divided into three groups according to the relative proportions for different time-of-day peaks as indicated in Figure 7 (a). Figure 7 (b) shows the distribution of rounds of dialogue between the users and physicians. For inquisitions, the number of rounds of dialogue peaked at around the *6 to 10* range; for consultations, at least half of the dialogues went through *0 to 5* rounds.

### Users’ Satisfaction With Online Consultations

**Figure 8:**
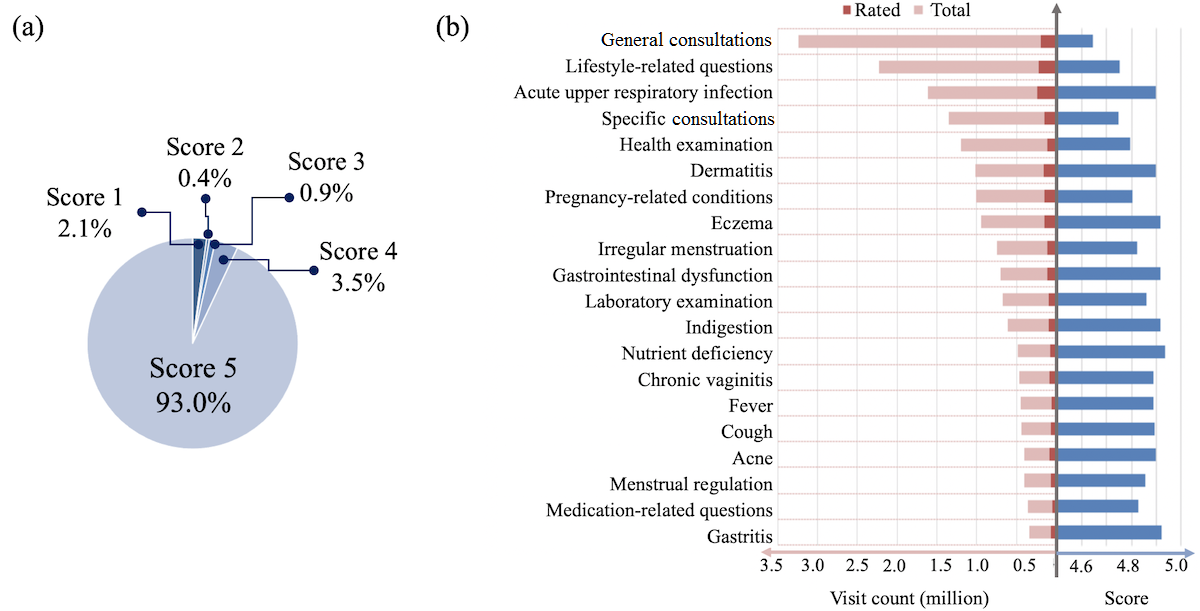
Users’ satisfaction with the online consultations: (a) the distribution of satisfaction scores; (b) the proportion of users who scored the consultations and inquisitions and the corresponding scores related to the top 20 diseases. Scores range from 1 (least satisfaction) to 5 (most satisfaction).

Among all the 35.3 million consultations, there were 5.2 million that had satisfaction scores. A total of 93.0% (6,408,143/6,879,529) of these satisfaction scores were 5, suggesting that the vast majority of the users had a good experience when using Ping An Good Doctor, as seen in Figure 8 (a). We also analyzed user satisfaction in terms of different diseases. As shown in Figure 8 (b), consultations related to the top 20 diseases all had average satisfaction scores above 4.6. For some specific diseases, the average satisfaction scores were even higher, such as for AURI and dermatitis, which were also popular topics of consultation in our internet hospitals. For *general consultations* and *specific consultations*, the satisfaction scores were relatively low.

## Discussion

### Principal Findings

Analyses in this study answered the three questions proposed in the Introduction section. First, the distributions of departments and diseases in Ping An Good Doctor differed from those of offline hospitals. The gynecology and obstetrics department and the dermatology department were the top two departments in Ping An Good Doctor in terms of number of consultations, which differed from offline hospitals. The popularity of gynecology and obstetrics came from about 22% of the users being female, aged 19 to 35 years, who were in the reproductive age group. The popularity of dermatology suggested that users found it suitable and more acceptable to seek consultations about dermatological diseases online. Consultations and diseases with moderate symptoms were commonly seen in terms of disease profiles. Second, we found that most of the users of Ping An Good Doctor were between 19 and 35 years of age, and females were slightly more highly represented compared to males. The gender distribution was in agreement with previous work by Qiu et al, though their results were based on one city with a much smaller population [34]. Last, the users had a good experience with Ping An Good Doctor, in general. We observed an average satisfaction score of 4.6 out of 5, suggesting that users were comfortable with Ping An Good Doctor as a health care service.

Our results provided the distribution profiles of departments and diseases. By examining those distributions, we could gain an increased understanding about which diseases can be diagnosed and treated online. The user profile analysis described the current user market of internet hospitals; a new strategy to explore the market should be based on this user profile analysis.

### Open Attitudes Were the Key Factor for Developing Internet Hospitals

Based on the geographical distribution of the Ping An Good Doctor visits, we found that Shandong, Yunnan, Shaanxi, and Guangdong were the four leading provinces in terms of volume of visits; the reasons for this were four-fold. First, Shandong and Guangdong have large populations. Second, high-quality health care services are limited in these four leading provinces; Shandong, Yunnan, Shaanxi, and Guangdong ranked 17^th^, 24^th^, 20^th^, and 19^th^, respectively, in terms of number of tertiary hospitals per 100,000 persons, according to the China Health Statistics Yearbook 2020 [13]. Third, Guangdong and Shandong have a relatively high internet penetration rate [35]. Fourth, open attitudes toward internet technology were the key factor for developing internet hospitals. Guangdong was the pioneer in online health care services. The Guangdong government funded the first internet hospital in China [14]. Shandong has the most online physicians in a web-based medical consultation service in China, which suggests an open mind on the part of the physicians [36]. The Yunnan and Shaanxi governments also have an open attitude toward the internet-related industry and the public has a high acceptance of internet technology.

It was interesting to note that the usage of internet hospitals was not centered in big cities. For example, Beijing and Shanghai ranked 9^th^ and 5^th^, respectively, among all the cities, although these two are megalopolitan cities with populations of more than 20 million. Kunming, the capital of Yunnan province with a population of around 7 million, had more yearly visits than Shanghai. The geographical distribution of yearly visits to Ping An Good Doctor suggested that internet hospitals provided accessible health care services to anyone in need and could relieve the uneven distribution of health care services across China.

### Nondisease-Specific Consultations and Moderate Health Problems Were Popular in the Top Five Departments

The top five departments represented in Ping An Good Doctor were gynecology and obstetrics, dermatology, pediatrics, general internal medicine, and urology and andrology. As the characteristics varied across departments, we will discuss them one by one.

In gynecology and obstetrics, the most common consultations and inquisitions were focused on menstruation, vaginitis, and cyesis. These were mild health issues as compared with those in offline hospitals where cancers; cervical disease, such as hysteromyoma and ovarian cyst; and obstetric complications were seen in higher proportions. These profiles were consistent with the highest ratio of users who sought consultations in gynecology and obstetrics being aged 19 to 35 years, as seen in Figure 6 (b).

For dermatology-related concerns, the most common diseases were dermatitis and eczema, as seen in Figure 5 (b). The same pattern was also reported in other work [37]. It was interesting to note that disease inquisitions played a major role as compared with consultations. The most important reason for this was that the online health care service could replicate the offline clinical scenarios to the largest extent. Users were able to upload pictures of their skin, which played a vital role in diagnoses and treatments in the dermatology department. In addition, the efficiency and timeliness of online health services encouraged users to choose online inquisitions when they had dermatological problems. Online consultations also allowed patients to avoid the potential embarrassment of exposing themselves to physicians face-to-face.

In pediatrics, the most common inquisitions were about AURIs as well as growth and development, such as nutrient deficiency. Recent work on a pediatric map of Hangzhou also found that AURI was the top disease discussed in all the outpatient visits they considered. However, the next most popular category of diseases in their study was common symptoms, such as fever, abdominal pain, and vomiting, which was distinct from the pattern we found in the online health service [38]. Internet hospitals provide a way to efficiently and conveniently acquire knowledge about children’s growth and development, relieving parents’ anxiety. Educated with knowledge about their children’s growth and development, parents can make proper and timely decisions when needed. Mild medical cases were able to be resolved online, which reduced unnecessary visits to offline hospitals and also relieved the stress experienced by them.

The diseases that were part of the general internal medicine department were mostly chronic, indicating that health management was in high demand. This was in agreement with previous findings from Di Tommaso et al [39]. Meanwhile, health management was vital for reducing fatality rates and disability rates. The proper management of chronic diseases could reduce the occurrences of complications and acute episodes. Traditionally, physicians mainly focus on chronic diseases at the time of patient in-person, offline visits; they have no time to conduct daily management for patients with chronic diseases. With developments in artificial intelligence and wearable devices, health care data are easy to collect and analyze. With well-educated patients and automatic tracking of physical measures, smart health care management has relieved physicians from tedious work and helped patients to maintain clinical conditions. Meanwhile, patients can benefit from advice on diet, exercise, and medication as given by smart health care management.

In the urology and andrology department, the consultations and inquisitions were focused on sexual function and urinary system infections. The online consultations were private and allowed users to avoid the embarrassment of face-to-face inquiries about sexual function problems. Urinary system infections were also common in both online and offline hospitals, but other frequent diseases seen in offline hospitals, such as cancer, urinary stones, and surgical trauma, were uncommon in online consultations.

To summarize, the common diseases seen in internet hospitals included moderate health problems. In this sense, internet hospitals play the role of general practitioner, which partially fills a gap that exists in the family doctor system in China.

### Relationship Between the COVID-19 Epidemic and Internet Hospitals

The COVID-19 epidemic promoted widespread use of internet hospitals in China. From Table 4 and Figure 4, we observed that consultations and inquisitions did not decrease even after the first outbreak of COVID-19, from January 23, 2020, to February 29, 2020. COVID-19 provided people with an opportunity to change their behavior when seeking health care services. With the epidemic under control, the development of internet hospitals has normalized [40]. On the other hand, internet hospitals played a vital role in China's defense against COVID-19, from sharing knowledge on virus control and providing health consultations to follow-up treatment for chronic diseases and drug delivery. Ping An Good Doctor also played a positive role during the COVID-19 pandemic, suggesting that internet hospitals are a valuable resource in dealing with large-scale public health emergencies [41].

### More Advantages of Internet Hospitals

In addition to the pros mentioned above, internet hospitals had many other advantages. First, internet hospitals had a positive effect on the physician-patient relationship [42]. Online health services made health information more accessible to patients, reducing the communication barrier between physicians and patients.

Second, for the physicians, providing health care services via internet hospitals was a way to not only increase their income but to widen and deepen their clinical experience of patients. The patients they met were not limited to their local regions. Meanwhile, physicians had the opportunity to inspect the whole disease process for the patients, from the onset to the prognosis.

Finally, internet hospitals could accelerate the reform of government health care insurance in China and possibly the world. Medical insurance costs increase year by year. The total cost reached 2085.4 billion yuan (US $299 billion) in 2019 in China, and had increased by 12.2% compared to 2018 [43]. Internet hospitals could help control four aspects of medical insurance costs. First, because the process of consultations and inquisitions was documented and all data were traced, it is feasible to conduct quality control and fraud detection. Second, equipped with artificial intelligence, standardized diagnosis and treatment procedures were simplified; for example, a robot helps to collect basic information before the consultations begin. Therefore, with increased efficiency, the cost of consultations in internet hospitals would decrease. Third, the whole process of health management in internet hospitals controls the rate of chronic diseases and, in the end, reduces medical insurance costs. China has a large population living with chronic diseases. Until 2019, there were 116.4 million people with diabetes in China [44]. The glucose control rate was 10.2% in China and was 49.9% in America [45]. A higher control rate is related to slower disease progression, lower comorbidity risk, lower disability rate, and lower costs. Efficient health management could help to increase the control rate and reduce medical costs. Fourth, internet hospitals could promote the separation of medical services and medications, which would prevent doctors from receiving rebates from drugs.

### Limitations

We described the characteristics of internet hospitals in terms of the distributions of departments, diseases, user profiles, user behaviors, and user experiences. However, treatment—an important aspect of clinical practice—was not investigated and evaluated, but will be targeted in our future work. To deepen our understanding of the characteristics of internet hospitals, our future efforts will include additional comparisons between offline hospitals and internet hospitals.

### Conclusions

As the largest entry point to online medical services in China, Ping An Good Doctor provides accessible health care services nationwide, breaking the geographical boundary for high-quality clinical resources. The COVID-19 epidemic accelerated the expansion of online health services. This also provided an opportunity to test the feasibility of such online modes of health care delivery. It should be emphasized that after the outbreak of COVID-19, the number of daily visits kept increasing, suggesting that online health services satisfied peoples’ needs for convenient and contactless health services. Our analyses showed that nondisease-specific consultations and moderate health problems accounted for the majority of online visits to Ping An Good Doctor. This indicated that online health services played a similar role as family doctors. The positive feedback received from most users showed that online health services would be their preferred option in many circumstances. The changes in people’s attitudes and behaviors, together with support from the government, would further promote the development of online health services, leading to great benefits for both patients and health care providers.

## Abbreviations

AURI: acute upper respiratory infection
ICD-10: International Classification of Diseases, Tenth Revision
TCM: Traditional Chinese Medicine
